# Explicit Logic Circuits Discriminate Neural States

**DOI:** 10.1371/journal.pone.0004154

**Published:** 2009-01-07

**Authors:** Lane Yoder

**Affiliations:** Department of Mathematics, University of Hawaii, Kapiolani, Honolulu, Hawaii; University of Southern California, United States of America

## Abstract

The magnitude and apparent complexity of the brain's connectivity have left explicit networks largely unexplored. As a result, the relationship between the organization of synaptic connections and how the brain processes information is poorly understood. A recently proposed retinal network that produces neural correlates of color vision is refined and extended here to a family of general logic circuits. For any combination of high and low activity in any set of neurons, one of the logic circuits can receive input from the neurons and activate a single output neuron whenever the input neurons have the given activity state. The strength of the output neuron's response is a measure of the difference between the smallest of the high inputs and the largest of the low inputs. The networks generate correlates of known psychophysical phenomena. These results follow directly from the most cost-effective architectures for specific logic circuits and the minimal cellular capabilities of excitation and inhibition. The networks function dynamically, making their operation consistent with the speed of most brain functions. The networks show that well-known psychophysical phenomena do not require extraordinarily complex brain structures, and that a single network architecture can produce apparently disparate phenomena in different sensory systems.

## Introduction

The relationship between the organization of synaptic connections and how the brain processes information is poorly understood. For some reflex responses the connectivity has been discovered by direct observation, and some theoretical networks have been proposed to explain other simple neural responses, e.g., [1]–[5].

The explicit networks introduced here are formal logic circuits that can discriminate degrees of state, and combinations of degrees of state, of any number of neurons. This is different from mathematical models that characterize and manipulate that information, and different from implicit networks that depend on assumed capabilities of unspecified component networks. As such, these networks may provide a more tangible model for how information is processed by the brain.

The following exposition first illustrates the difficulty of the task by describing what is required to interpret the information contained in several olfactory sensor cell responses to a mixture of odorants. Then logic circuits are described that can effectively summarize the sensory information and provide for perceptual and cognitive distinctions. Although many models have been proposed for neuronal encoding of information, only the minimal, known cellular capabilities of excitation and inhibition are needed to derive the network properties. A neuron with excitatory and inhibitory input is shown to function as a simple logic gate. Several of these logical primitives are connected to form general logic circuits that can perform negations and conjunctions of any number of propositions. The architectures of these circuits are different from the standard architectures of electronic logic circuits in several ways. In addition to the classical logic of discrete true and false values, these logic circuits perform fuzzy logic operations that can deal with degrees of certainty. This is a powerful tool in processing ambiguous or incomplete information.

To demonstrate the capabilities of these logic circuits, they are shown to generate neural correlates of complex psychophysical olfactory phenomena for mixtures and varying concentrations of odorants. To illustrate the general nature of the networks' capabilities, the networks are shown to produce identical phenomena for color vision. This demonstrates that the networks' transformation of input data provides basic information processing for the perceptual and cognitive operations of the brain. In conclusion, the networks' differences from the brain and from other models of brain function are discussed.

## Analysis

### Odor Discrimination

In a classic series of experiments, it was shown that each olfactory receptor cell expresses exactly one odorant receptor gene that produces one type of odorant receptor [6]. Humans have been found to have 388 classes of olfactory receptors [7]. The exact number is not critical here. Each of these cell types responds with varying sensitivity to several related molecules, and cells in different classes have different sensitivities. Each odorant molecule activates several classes of olfactory cells with varying intensity. Most odorants are composed of several odorant molecules. The particular combination of odorant molecules in each odorant induces a corresponding combination of olfactory receptor cell responses that are transmitted to the brain.

Consider the problem of discriminating odors based on the signals from receptor cells. To take a simple example, suppose an odorant is composed of six different types of molecules, and each of six olfactory receptor types is sensitive to one of these molecules. Some of the more obvious possible models for discriminating the odorant have significant problems. Perhaps the simplest model would produce a different sensation for the response of each receptor type. Providing a perception of each component of a pattern does not provide a basis for identifying the pattern; it merely shifts the pattern, and the problem of identifying it, elsewhere in the brain. Another possibility is to connect a neuron to the receptors in such a way that it is activated if and only if all six of the receptor types are active. This model does not take the odorant's concentration into account. Since each olfactory receptor cell responds with varying sensitivity to several related molecules, a few odorant molecules to which a receptor is highly sensitive can elicit the same response as many molecules to which it is only slightly sensitive. Without some means of separating the effect of the type of molecule from the effect of concentration on receptors' responses, information about the odorant's identity will remain conflated with concentration.

Still another mechanism could normalize the strengths of the six responses to correct for different odorant concentrations and then compare the normalized responses to a stored set of values representing the particular odor. This model has computational obstacles. Although normalizing several values is a simple process mathematically, connecting logic gates to implement such a procedure is not, regardless of the kind of hardware used. The same is true of the other two processes – comparing several values and arriving at a single conclusion based on the comparisons. Moreover, nonlinearities in neural response functions greatly increase the complexity of the task. Although the brain is capable of such computationally intensive operations, the required logic circuits would be quite large and complex.

A far more difficult problem than the above example is discrimination of the individual odors in a mixture of odorants. To the extent that such discrimination is possible, a biologically adaptive olfactory system should have this ability. The patterns of receptor responses produced by odorant mixtures can be quite complex, especially when two or more of the components of a mixture contain some of the same or similar molecules that activate the same receptor types. Even a mixture of just two odorants can elicit a wide variety of receptor responses when both the proportion of each odorant and the concentration of the mixture are varied. Identifying the component odorants is correspondingly challenging.

One of the basic concepts of information theory is that the smaller the probability of an event, the more information it contains. This means that if a particular molecule is present in most odorants, its absence holds more information about an odorant's identity than its presence. For example, suppose an olfactory receptor type X is sensitive to a molecule that is present in all but four odorants. Signals from one or more receptor types but not from X narrow the identity of an odorant to four possibilities. An odorant that does not contain the common molecule has such a small probability, and therefore conveys so much information, that just two additional bits of information are necessary to identify the odorant among the four possibilities. Even if a molecule is present in only a few odorants, its absence still carries some information about an odorant's identity.

Because of the information contained in null responses, the set of receptor types that are best suited to identify an odorant may contain receptor types that are not sensitive to the odorant as well as types that are. As a simple example, suppose an odorant is the only odorant that elicits relatively high intensity responses from receptor types X_1_ and X_2_ and relatively low intensity responses from types X_3_ and X_4_. Discrimination of the odorant can be based on some measure of the extent to which all four of these conditions are satisfied. One possibility is a neuron whose response intensity is a measure of the difference between the smaller of the responses from receptors X_1_ and X_2_ and the larger of the responses from X_3_ and X_4_.

### Neuronal Encoding of Information

Deriving a network's behavior requires some assumptions about the behavior of the network's components. Many models have been proposed for neuron responses. Most models fall into one of two categories. One is the pulse model, such as the model of McCulloch and Pitts [8] and the integrate-and-fire model. The second is the firing rate model, such as that proposed by Hopfield [9]. A review of these and other models is provided by Koch [10]. A general problem with all models, as Hopfield [9] pointed out, is that it is uncertain whether the assumptions hold for real neurons. The more detailed the assumptions are, the greater the uncertainty.

To take just one example, a standard model for neuron response assumes that activation is a nonlinear function of a weighted sum of the inputs. This function may appear to be fairly general, but it cannot express quite simple functions of two or more variables or even produce reasonable approximations of them. For example, a possible neuron response to excitatory inputs X and Y is R[S(X)+S(Y)], where S is a sigmoid function that amplifies large inputs and reduces small ones, and R restricts outputs asymptotically below some physical bound on a neuron's maximum response. Because of the nonlinearity of S, S(X)+S(Y) cannot be expressed as a weighted sum of X and Y. This implies the response function R[S(X)+S(Y)] cannot be expressed as a nonlinear function of a weighted sum of X and Y.

Neuron responses to single excitatory inputs have been shown to amplify the effect of large inputs and reduce the effect of small inputs [11], [12]. This response property acts as a natural noise filter for low-level noise that is added to high or low intensity signals. If a neuron's input channel is carrying no signal, low-level noise in the channel is reduced or eliminated by such a response function. If an input signal that is near the maximum intensity is decreased by additive noise, amplification reduces or eliminates the effect of the noise. For more than one input, a noise filtering property for each of the inputs would be a reasonable evolutionary design. In that case, the standard response model that assumes a nonlinear function of a weighted sum of the inputs would not be appropriate. A linear weighting function for each input does not filter noise. Nor can the nonlinear function of the weighted sum effectively filter additive noise. This is because the weighted sum of low-level noise in several input channels can be large enough to be indistinguishable from a strong signal in one of the input channels.

Minimal, known characteristics of cellular behavior are sufficient to show the networks presented here generate known phenomena. Some types of neurons and sensory receptors have graded responses rather than all-or-nothing action potentials. For most types, however, the intensities of the input and output can be measured, and the most basic relationship between input and output is known: The intensity of an external stimulus is reflected in the sensory receptor's response, and this intensity is carried over in the signal's transmission from receptor to neuron and from neuron to neuron. Cells whose responses consist of action potentials are generally thought to convey the intensity in the frequency of action potentials. Table 1 lists these most basic properties of cellular signals in somewhat more detail. The response intensity is assumed to be measured at some moderate level of adaptation. Phenomena associated with adaptation are not considered here, so a constant level of adaptation is assumed. For convenience, response intensities are normalized by dividing them by the maximum possible intensity for the given level of adaptation. This puts response intensities in the interval from 0 to 1, with 0 meaning no signal and 1 meaning the maximum intensity. This number will be called the *response intensity* or simply the *response* of the receptor or neuron. If a neuron's response is not 0, the neuron is said to *respond*. Normalizing the responses does not affect any of the results derived here. To avoid confusion, perhaps it should be noted that for cells that transmit all-or-nothing action potentials, the response, as defined here, is the normalized frequency of action potentials, not a measure of the membrane potential or how near the potential is to the threshold for an action potential.

**Table 1:** Cellular response properties.

1. 1∼0 = 1.	Maximum excitation elicits maximum response.
2. X∼Y = 0 if X≤Y.	Inhibition cancels equal or smaller excitation.
3. X∼Y is increasing in X.	Greater excitatory input increases output.
4. X∼Y is decreasing in Y.	Greater inhibitory input decreases output.
5. Olfactory receptor response is an increasing function of odorant concentration.	
6. Photoreceptor activity is a decreasing function of photostimulus intensity.	

The properties of the neural logic circuits follow from the networks' architectures and the minimal, well-known cellular characteristics listed here. If X and Y are two cells' response intensities, X∼Y represents the response intensity of a neuron with excitatory input X and inhibitory input Y. Responses are normalized to be in the interval from 0 to 1.

Most neurons in the cortex either excite or inhibit other cells, but not both. Excitatory cells can inhibit by exciting an inhibitory cell. If the responses of two sensory receptors or neurons are X and Y, the notation *X∼Y* will represent the response of a neuron with excitatory input X and inhibitory input Y. The properties in Table 1 that say the response X∼Y is an increasing or decreasing function refer to the fact that input and output intensities are variables; the response is not increasing or decreasing with time. Although Table 1 is used to derive the various networks' properties, it is not part of the models. The models are defined by the figures.

For the conclusions of this article to hold for networks constructed with real cells, the minimal properties of Table 1 only need to be approximations of actual complex neuron responses. Little information is available for the actual behavior of the neuron response function X∼Y, for example. Property 2 may actually be X∼Y = 0 if g(X)≤Y for some function g(X) that only loosely approximates the identity function I(X) = X. Such minor adjustments in the properties of Table 1 would modify the conclusions about the networks only by degrees; they would not negate the conclusions.

### Neural AND NOT Gates

A few elementary concepts of classical logic are needed here to show that neurons can function as logic gates. The customary logic notation will be used. Variables X and Y represent truth values of propositions. The value 0 stands for false, and 1 stands for true. The notation XY stands for the value of the conjunction X AND Y (X and Y are both true), 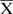 stands for NOT X (X is not true), and 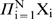 stands for X_1_X_2_…X_N_ (all X_i_ are true). These logic functions have the customary truth values: XY = 1 if and only if X = Y = 1, 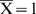 if and only if X = 0, and 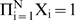 if and only if X_1_ = … = X_N_ = 1. For the recursive logic identities given in the next section to be true, 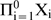 is defined to be 1. For now, neuron responses are assumed to be either 0 or 1 to coincide with the values of classical logic. Intermediate neuron responses between 0 and 1 will be considered later. A neuron's response can represent the truth value of a proposition. For example, the response of an olfactory receptor cell can be interpreted as the truth value of “the receptor cell is activated.”

**Figure 1:**
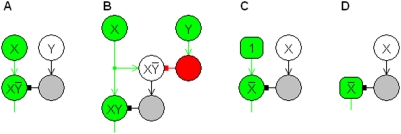
Neurons as functionally complete logic gates. The circuit diagrams show that neurons with excitatory and inhibitory inputs and neurons that have continuously high outputs form a functionally complete set, meaning any logic circuit can be constructed with them. The label on each neuron represents its response. The maximum and minimum possible responses 1 and 0 can stand for the logical values true and false, making the network outputs logical functions of the inputs. The diagrams show logic gates for (A) X AND NOT Y, (B) X AND Y, and (C, D) NOT X. Arrows indicate excitatory input; blocks indicate inhibitory input. Spontaneously active neurons are square. To illustrate example inputs and outputs, active neurons are colored. Inactive inhibitory cells are shaded.

Property 1 of Table 1 says the cellular response X∼Y is 1 if X = 1 and Y = 0. By property 2, X∼Y is 0 for the other three possible combinations of X and Y values. The logical conjunction 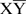 (X AND NOT Y) has the same truth values. This means the neuron performs the logical AND NOT function: 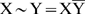. A neuron with one excitatory input and one inhibitory input will be called a *neural AND NOT gate*. Its response property 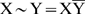 is the *neural AND NOT property*. This logic gate is illustrated in the circuit diagram in Fig. 1A. To illustrate example inputs and outputs, active neurons are colored in the figures. Inactive inhibitory cells are shaded. The cells shown providing input to the networks in the figures are not considered part of the networks and need not even be near the networks. They could be sensory cells or the output cells of other networks.

For networks consisting of neural AND NOT gates, their outputs can be determined either by the properties of Table 1 or, usually more conveniently, by the neural AND NOT property and the algebra of classical logic. For example, X∼(X∼Y) denotes the response of a cell that has excitatory input X and inhibitory input X∼Y, as illustrated in Fig. 1B. This response is the logical AND function XY because 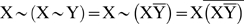 by the neural AND NOT property, and 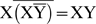 by the algebra of classical logic. It might appear that the function X AND Y could be achieved simply by a cell with two excitatory inputs, X and Y, but this cell's output would be the logical function X OR Y since a high value of either X or Y would activate the cell.

Fig. 1C shows another simple but important logic circuit known as an inverter. If a neural AND NOT gate has inhibitory input X and excitatory input from a neuron whose response is constantly 1, then the output 1∼X is the logical NOT function 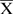 because 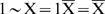. A response that is constantly 1 could be provided by a special purpose neuron that fires spontaneously and continuously. Such neurons are known to exist in the brain [13]. They keep people awake, for example, and sleep apparently requires inhibition of these neurons [14]. Alternatively, the NOT cell itself could be a spontaneously active neuron with only inhibitory input, as illustrated in Fig. 1D. In the figures, spontaneously active cells are square to distinguish them from other cells. Any spontaneously active cell could be replaced by an ordinary cell with continuous excitatory input. A single spontaneously active neuron could provide continuous excitatory input to several ordinary cells, making them function the same as spontaneously active cells.

An AND gate and a NOT gate are logical primitives that together make up a “functionally complete” set, meaning for every possible logical proposition there is a way to define it in terms of the members of the set. For a complex logical proposition, however, such a definition may not be obvious, and a definition that provides an efficient architecture for implementing a logic circuit for the proposition is likely to be even more obscure. Fig. 1 shows that both AND gates and NOT gates can be constructed from AND NOT gates and gates that have continuously high output. This means that a neuron with excitatory and inhibitory inputs and a neuron that has a continuously high output are logical primitives that make up a functionally complete set. With one minor exception, this article's networks consist of neural AND NOT gates and spontaneously active neurons.

Functionally complete components are especially significant when they are available in large numbers, and the abundance of neurons is the main distinguishing feature of the human brain. Given any function that can be executed by a computer, for example, and given enough functionally complete components, there is a way to construct a logic circuit that performs the function. This also applies to any set of functions that any number of computers could conceivably perform, with parallel or sequential processing, including everything computer software could possibly do because any software can be implemented in hardware.

The logic circuits presented here differ from electronic logic circuits in several ways besides the obvious differences in the physical natures of their components. Logical AND NOT gates are not common in electronic systems. Electronic logic circuits are often implemented with NOR (not or) or NAND (not and) gates because these gates are relatively economical to produce with transistors and semiconductor diodes and because they are sole sufficient operators, meaning each is functionally complete by itself. Because of this difference, the architecture of the logic circuits presented here is likely to be new. Each of the logical primitives NOR and NAND requires several electronic components, whereas one neuron with a second neuron providing inhibitory input can function as a neural AND NOT gate. Although it would be possible to construct NOR and NAND gates from neural AND NOT gates and spontaneously active neurons, and then use these components to assemble logic circuits with the standard architecture found in logic design textbooks, the resulting networks would be needlessly complex and require far more neurons than necessary. A property discussed later is that neurons can have responses of varying intensity, while most electronic logic gates encode information in discrete zeros and ones. It will be seen that this neuronal capability is a powerful tool in processing information. Finally, it will be shown in a future paper that logic circuits formed with AND NOT gates require no more component gates than standard electronic logic circuits using NOR or NAND gates.

### Recursive AND NOT Conjunctions

The networks presented here are general logic circuits that can perform logical negations and conjunctions of any number of propositions. Logical conjunctions determine whether or not several conditions are satisfied. Much of the brain's information processing involves such decision making, from controlling breathing, heart rate, and balance, to discerning form, movement, and faces, to producing the creative and analytic thought processes involved in reasoning, planning, and decision-making. For example, a photostimulus is perceived as green when the M cone (that is sensitive to medium wavelengths of light) has a high absorption of photons and the S and L cones have low absorptions. The compound proposition “M has a high absorption, and S has a low absorption, and L has a low absorption” is a conjunction of three propositions. A neural correlate of the perception of green is the response of a neuron that is activated when the conjunction is true.

The logic identities in the first column of Table 2 show that every conjunction is logically equivalent to a single AND NOT conjunction 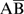. To make them clear, A and B are enclosed in braces. These logic identities are easily verified by the algebra of classical logic. The identities are recursive and reductive. Equations 1 and 2 say that a conjunction of n = M+N propositions is logically equivalent to 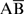, where A and B are each conjunctions of n – 1 propositions. By the same equations, each of the conjunctions A and B is equivalent to the conjunction of two propositions that are each conjunctions of n – 2 propositions, and so on, until A and B are two of the propositions X_i_ and Y_j_. Equations 3 and 4 say a conjunction of N propositions is equivalent to a conjunction 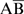, where A is a conjunction of N – 1 propositions and B is a conjunction of N propositions. Proposition A can be further reduced by equation 3 or 4, and B can be reduced by equation 1 or 2.

**Table 2:** Recursive AND NOT Conjunction definitions and responses.

Recursive logic identity for constructing a RANC	Interval measured by the RANC response	Approximate value of the RANC response
1. 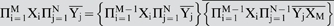	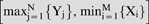	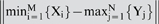
2. 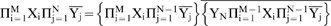		
3. 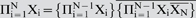	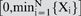	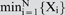
4. 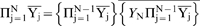	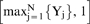	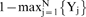

The logic identities in the first column equate every conjunction to a conjunction 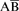. The recursive and reductive identities show how logic circuits can be implemented with neural AND NOT gates using the neural AND NOT property 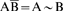. The second column shows the interval measured by the corresponding RANC response. The third column shows the approximate value of the response is the length of the interval. The notation ∥b−a∥ stands for the length of the interval [a, b] if a<b, and 0 otherwise.

Here the significance of recursively equating every conjunction to 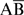, where A and B are reduced conjunctions, is that it shows how conjunctions can be implemented entirely with neural AND NOT gates by repeated use of the neural AND NOT property 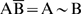. For example, if M = 2 and N = 1, equation 1 says 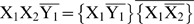. Applying the neural AND NOT property three times, this can be implemented as 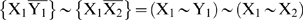. Substituting X_3_ for Y_1_ gives 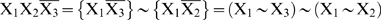. This network will be seen shortly in the next figure.

If M = N = 1, equations 1 and 2 in Table 2 both reduce to 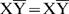, and this conjunction is implemented as X∼Y. For more inputs, equations 1 and 2 show two different ways of implementing the same conjunction. If N = 1, equation 1 requires fewer neurons and retains a negated component in both A and B, 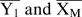 respectively. Similarly if M = 1, equation 2 requires fewer neurons and retains a non-negated component in both A and B, 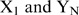 respectively. The implementation that has fewer neurons also has the important property of measuring differences between inputs. This property is discussed later with intermediate input values. If M and N are both greater than 1, either implementation can be used. The two resulting networks are different but have the same architecture and the same number of neurons. Although equations 1 and 2 work equally well for implementing a single conjunction, they are used in an alternating pattern here to obtain the most efficient architecture for several conjunctions.

A conjunction logic circuit that is constructed from AND NOT gates according to the recursive logic identities of Table 2 is a *Recursive AND NOT Conjunction* (RANC). For n propositions that have one of two possible truth values, true or false, there are 2^n^ possible combinations of truth values. Each combination corresponds to a conjunction of n propositions. For two propositions X and Y, for example, there are four conjunctions: 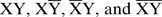. An *n-RANC* produces one or more conjunctions of n propositions. A *single* n-RANC produces one of the conjunctions, and a *complete* n-RANC produces all of the 2^n^ possible conjunctions.

**Figure 2:**
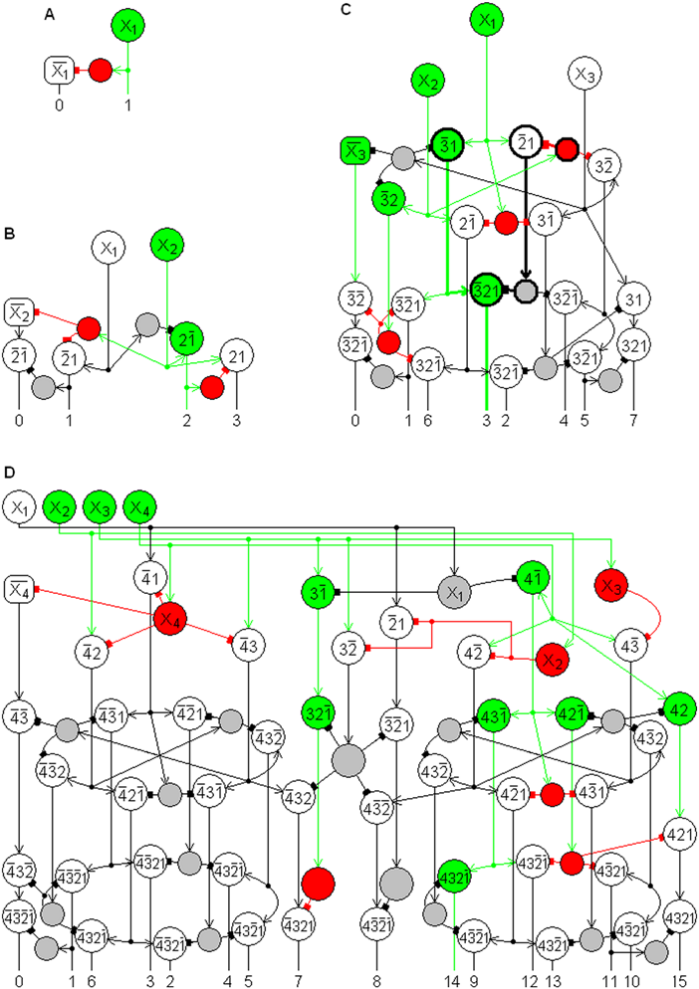
Recursive AND NOT Conjunctions. An n-RANC is a general logic circuit that produces conjunctions of n propositions. A complete n-RANC produces all conjunctions corresponding to the 2^n^ possible combinations of truth values of n propositions. Examples of complete n-RANCs are shown here for n = 1-4. A single n-RANC produces one of the possible conjunctions. In C, the single 3-RANC that produces output number 3, 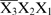, is indicated by thick lines. In D, the output number 14, 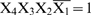, represents the truth value of the conjunction “X_2_, X_3_, and X_4_ are high, and X_1_ is not high.” The other 15 conjunctions are false, and the corresponding RANC outputs are 0.

Examples of complete n-RANCs are shown in Fig. 2 for n = 1-4. The illustrations are three-dimensional because the networks' geometric properties of simplicity and symmetry that can be achieved in three-dimensional space are not apparent in conventional two-dimensional circuit diagrams. The views are exploded to show the cells and their connections clearly. Optimal geometrical configurations will be considered in a future paper. All of the RANCs are in columnar structures. The columns could be oriented in any direction (as are cortical columns), but in the figures they are oriented with a vertical axis and the outer layer at the top to agree with customary depictions of the cortex. As in Fig. 1, active cells are colored in Fig. 2 to illustrate example responses.

For subscripted conjunctions, only the subscripts are shown. For example, the response 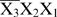 is abbreviated as 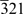. In this abbreviated notation, the earlier example 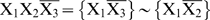 is written 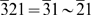. The cells with these abbreviated labels are illustrated with thick lines in Fig. 2C. The subscripts are written in descending order to match the standard digit ordering of the numeric labels for the networks' outputs. For example, the binary number 011 equals the decimal number 3 (0(2^2^)+1(2^1^)+1(2^0^) = 3). The response 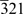 in Fig. 2C is labeled “3” below the network because it has the value 1 if and only if the inputs X_3_, X_2_, X_1_ are 0, 1, 1, respectively. This particular state of RANC cell responses is illustrated by the colors in Fig. 2C. The whole number labels for the RANC outputs are meant to provide a short and mnemonic way of referring to the different outputs, but the labels should not be confused with the variable output values.

### Fuzzy Logic

It was shown above that RANCs are logic circuits that produce the conjunctions of classical logic when the inputs have the binary values of 0 and 1. However, neurons normally respond with a variety of intensities that reflect the intensities of their inputs. This raises the question of what function the RANCs produce for intermediate inputs. It turns out that RANC outputs are a generalization of standard fuzzy logic truth values, and this RANC fuzzy logic has biologically adaptive properties and generates correlates of brain phenomena.

The field of fuzzy logic was developed expressly to mimic the human brain in making decisions based on information that is ambiguous, imprecise, or incomplete [15]. Since this is the kind of data the brain receives from its sensory systems, some kind of fuzzy logic is virtually a necessity for the brain to cope successfully with the external world. In fuzzy logic, propositions such as “It's cold” and “The car is moving fast” can have intermediate degrees of truth between absolutely false and absolutely true. That is, the truth value can be any number in the interval [0, 1]. Fuzzy logic has been successful in a variety of practical applications, especially in Japan and Europe. Ironically, how neurons perform logical functions using intermediate information states remains virtually unknown.

Applications of fuzzy logic are normally implemented on electronic processors, and nearly all electronic logic gates encode information in high and low values, i.e., 0 and 1, simply because that is the most efficient and reliable way of processing information with electronic components. This means all numbers, including decimal fractions, are encoded in sequences of zeros and ones. That method of encoding numbers makes implementation of fuzzy logic computationally intensive. Neurons, of course, are different. Since a neuron's response can be any number in the interval [0, 1], it can represent the fuzzy truth degree of a proposition. Implementation of fuzzy logic with neurons is therefore much more efficient.

A truth function defines the truth value of a proposition composed by connectives (conjunction, disjunction, negation) in terms of the truth values of its components. One of the central questions in fuzzy logic is how the truth functions of classical logic should be extended to include intermediate truth values. Any such truth function should agree with classical logic when the components have the binary values of 0 and 1, but many functions do this. The simplest and most commonly used fuzzy truth function for conjunction is XY = min{X, Y}, the smaller of X and Y. The intuitive rationale for this function is that the proposition XY should have a high truth value if and only if both X and Y have high truth values. For negation, the most common function is 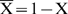. The intuitive rationale is that the proposition 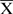 should have a high truth value if and only X has a low truth value. The next paragraph shows that the functions in the third column of Table 2 extend these standard fuzzy truth functions for conjunction and negation to conjunctions of any number of components with any of the components negated. The next section shows that RANCs produce a generalization of this fuzzy logic.

The second column in Table 2 contains intervals [a, b]. The third column contains the lengths of the corresponding intervals in the second column. The notation ∥b−a∥ in the third column stands for the length of the interval [a, b]; that is, ∥b−a∥ = b−a if a<b, and ∥b−a∥ = 0 if a≥b. The interval lengths of the third column define a fuzzy truth function for the corresponding propositions on the left side of the logic identities 1-4 in the first column. These truth values are clearly consistent with classical logic when the components have the binary values of 0 and 1. The truth values also generalize the standard fuzzy truth functions of the preceding paragraph: If N = 2, the truth value of proposition 3 in the first column is X_1_X_2_ = min{X_1_, X_2_}; and if N = 1, the truth value of proposition 4 is 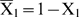. The truth values for propositions 3 and 4 are implied by the truth value for proposition 1 since 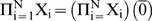 and 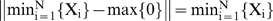; and 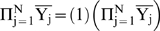 and 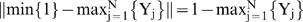. The intuitive rationale for the truth value for conjunction 1 is that 
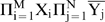
 should have a high truth value if and only if all X_i_ have high truth values and all Y_j_ have low truth values. The truth value 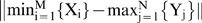 is consistent with this view.

### RANC Fuzzy Logic

Nonlinear neuron responses cannot produce the linear functions of the third column in Table 2. RANC responses do, however, come close to these values, and they convey just as much information. An important RANC property used frequently in the following discussions is that the neuron response for the conjunction in the first column of Table 2 is a measure (defined immediately below) of the corresponding interval in the second column. This will be referred to as the *RANC interval measure property*. The property can be proven by induction on the number of inputs, using the cellular properties of Table 1 and the RANC definitions of Table 2.

A mathematical measure, usually designated by μ, is a broad generalization of length. For the purposes of this article, a function μ[a, b] is defined to be a *measure* of any interval [a, b] if (1) μ[0, 1] = 1; (2) μ[a, b] = 0 if a≥b; and (3) μ[a, b]<μ[c, d] if [a, b]⊂[c, d]. By this definition, the length ∥b−a∥ is itself a measure of the interval [a, b]. Properties 1 and 2 in the definition imply that any measure of the intervals in the second column of Table 2 agrees with classical logic values for the conjunctions in the first column when the components have the binary values of 0 and 1. This means that any measure of the intervals in the second column of Table 2 is a fuzzy truth function for the conjunctions in the first column. Any such measure also generalizes the standard fuzzy truth functions for conjunction and negation.

By the definition of a measure, the RANC interval measure property says that a RANC response for a conjunction in column 1 of Table 2 increases from 0 to 1 as the endpoints of the corresponding interval in column 2 increase the interval length from 0 to 1. As before, this does not mean the response increases with time. If neuron responses were perfectly linear functions of their inputs, it can easily be shown from properties 1 and 2 of Table 1 that the RANC response would be equal to the length of the interval it measures. This implies the RANC response approximates the length of the interval, with the accuracy of the approximation depending on how close neuron response functions are to being linear. To the extent that neuron responses are approximately linear, the interval lengths in the third column of Table 2 can be taken as approximations of the corresponding RANC responses for the conjunctions in the first column.

The RANC interval measure property has two important consequences. First, for each of the possible orderings of the input intensities, such as 0 = X_2_<X_1_<X_3_<1, exactly one set of a complete n-RANC's output cells responds. That is, the combination of output cells that respond uniquely identifies the ordering of the input intensities. For the example above, the input ordering is 0 = X_2_<X_1_<X_3_<1 if and only if a complete 3-RANC has positive responses 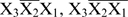, and 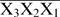. For another example, the input ordering is 0 = X_2_<X_1_ = X_3_ = 1 if and only if a complete 3-RANC has a single positive response 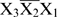. This identification of the input ordering is unambiguous in the sense that it is independent of the magnitude of the positive RANC output responses.

In general, the RANC interval measure property implies that a positive response 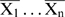 means all inputs are less than 1, and a positive response 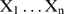 means all inputs are greater than 0. The positive output with the fewest negations in its response name indicates which inputs are smallest. If this output has more than one negated input, those inputs are equal. The positive output with the second fewest negations must contain the same negated inputs as the output with the fewest negations. The additional negated inputs are the second smallest inputs, and they are equal if there is more than one. Each additional positive output with the next fewest negations indicates the next smallest inputs.

Secondly, the input intensities partition the interval [0, 1] into subintervals, and the RANC response intensities are measures of the subintervals. The response intensity 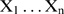 is a measure of the interval from 0 to the smallest input or inputs, the intensity of the positive output with the fewest negations is a measure of the interval between the smallest input or inputs and the next smallest inputs, and so on. The response 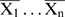 is a measure of the interval from the largest input or inputs to 1.

The RANC interval measure property also implies three more properties. Since n inputs partition the interval [0, 1] into at most n+1 subintervals and the RANC response intensities are measures of these subintervals, a complete n-RANC can have at most n+1 positive responses out of its 2^n^ responses. Since the RANC responses are approximately the lengths of the subintervals, the sum of a complete n-RANC's outputs is approximately 1. This bound on the outputs implies that not many of them can be large.

Functions of several variables, such as statistical functions, typically lose much of the information contained in the data. The two properties of RANC responses, identifying the input ordering and measuring the intervals formed by them, mean RANC responses retain all of the information in the inputs, but in a different form. The inputs could even be reconstructed from the outputs. The reconfigured information produced by RANCs is biologically adaptive and predicts known phenomena.

## Discussion

### RANC Fuzzy Logic Predictions of Olfactory Phenomena

#### Discriminating Odors

The fuzzy logic produced by RANCs effectively solves the problem of discriminating odors. Consider the earlier example of an odor that is perceived whenever the olfactory receptor responses X_1_ and X_2_ are relatively high and X_3_ and X_4_ are low. By the RANC interval measure property, the 4-RANC output 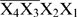 is a neural correlate of this perception. A moderate concentration of an odorant might elicit moderate receptor responses, say (X_1_, X_2_, X_3_, X_4_) = (0.4, 0.5, 0.0, 0.0). The response 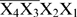 is a measure of the interval [max{X_3_, X_4_}, min{X_1_, X_2_}] = [0.0, 0.4], and the response is approximately its length, 0.4.

**Figure 3:**
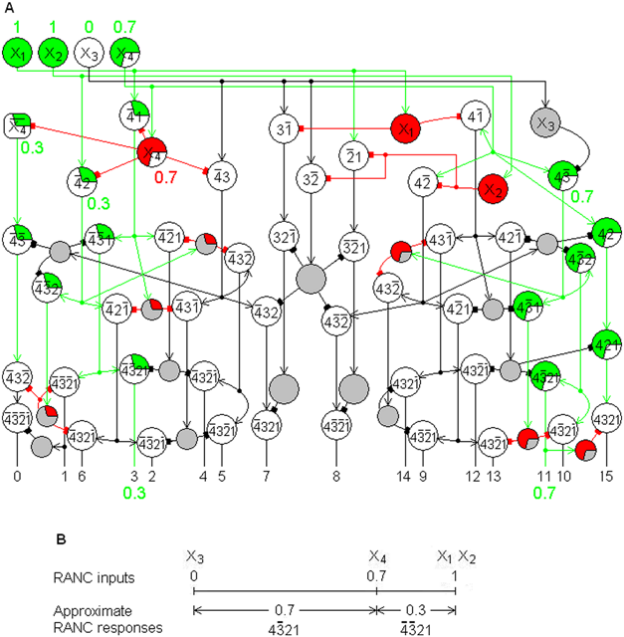
Fuzzy logic of a complete 4-RANC. The figure in A shows the approximate computations of a complete 4-RANC when one of the inputs has an intermediate value between 0 and 1. The graph in B illustrates the RANC interval measure property: The output intensities (approximately 0.7 and 0.3) are measures of the subintervals ([0, 0.7], and [0.7, 1]) of [0, 1] formed by the input intensities. The combination of output cells that respond 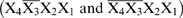 uniquely identifies the ordering of the input intensities (0 = X_3_<X_4_<X_1_ = X_2_ = 1). The response 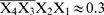 represents the fuzzy truth value of the conjunction “X_1_ and X_2_ are high and X_3_ and X_4_ are not high.”

The fuzzy logic of RANCs also discriminates individual odors in a mixture of odorants, even odorants that activate nearly identical sets of receptor types. Suppose a second odor is perceived whenever the receptor responses X_1_, X_2_, and X_4_ are relatively high and X_3_ is low. The output 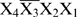 is a neural correlate of this perception. A certain concentration of an odorant could elicit the responses (X_1_, X_2_, X_3_, X_4_) = (0.8, 0.6, 0.0, 0.7). The response 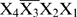 is a measure of [X_3_, min{X_1_, X_2_, X_4_}] = [0.0, 0.6], and the response is approximately 0.6. Since the third and fourth receptor types are relatively insensitive to the first odorant, a mixture of the two odorants could elicit responses (X_1_, X_2_, X_3_, X_4_) = (1.0, 1.0, 0.0, 0.7). In this case the same two RANC cells are activated, correctly identifying the component odorants, and their response intensities are 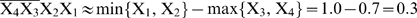 and 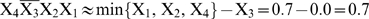. This state of RANC responses is illustrated in Fig. 3. Note that neither of these 4-RANCs responds to the wrong odorant. The response 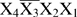 is 0 when the receptor states are (X_1_, X_2_, X_3_, X_4_) = (0.4, 0.5, 0.0, 0.0) because X_3_ = X_4_. Similarly 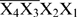 does not respond to (0.8, 0.6, 0.0, 0.7) because X_2_<X_4_.

If only one single n-RANC responds, that is the RANC correlate of a unique odor, i.e., one in which no more than one odor can be discriminated. A complete n-RANC has exactly one positive response if and only if all inputs have the binary values 0 or 1. Correlates of unique odors can also be produced by RANCs in several additional ways. Some single n-RANCs may not be present because they are not needed. For example, the output labeled 0, 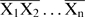, responds when there is no stimulus. Eliminating this single n-RANC would also eliminate the need for a spontaneously active neuron. Other single n-RANCs may not be needed because some combinations of receptor responses may not occur for any stimulus or because some combinations may be produced by substances that never had selective pressure for detection. If some single n-RANCs are not present, an odorant that would produce several responses in a complete n-RANC could elicit a single response from an incomplete n-RANC. Alternatively, suppose an odorant produces m outputs from a complete n-RANC, and suppose the order of the inputs from the receptors uniquely identifies the odorant. The m outputs identify the input ordering, which in turn identifies the odorant. The m outputs could be fed as inputs to a single m-RANC that produces 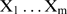. This cell responds if and only if all m inputs are positive. A response from this single cell indicates the input ordering that uniquely identifies the odorant. Finally, selective pressure to produce a unique response to a biologically important odorant could result in just the right sensitivity in several receptors so that the odorant produces just one response value in the receptors. For example, six receptor types responding to four different molecules in an odorant and two that are not in the odorant might have responses 0 = X_1_ = X_2_<X_3_ = X_4_ = X_5_ = X_6_. If the single 6-RANC for 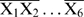 is not present, the only positive 6-RANC response is 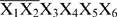.

#### Number of Odors that Can Be Discriminated

The RANCs show how the brain can discriminate a large number of odors in the different patterns of signals from a few hundred types of olfactory receptors. In some reports, humans can distinguish as many as 400,000 odors. This can be accomplished either by several RANCs that have input from a few sensor types or by a few RANCs with many inputs. For example, 388 receptor types can provide inputs to 27 distinct complete 14-RANCs. The total number of cells required is just over two million. Redundancies and other error-correcting mechanisms would of course require somewhat more cells. Each complete 14-RANC can have up to 2^14^−1 = 16,383 outputs, not counting the output labeled 0, for a total of 442,341 outputs. Each of these responses could be the neural correlate of a distinct odor.

The networks could be more complex than this model of distinct networks. Some single n-RANCs may not be needed, as was pointed out at the end of the preceding section. Some networks may overlap. For example, some single 10-RANCs might have inputs from receptor types labeled 1–10 for odors whose discrimination depends on various combinations of those receptor responses, and some single 14-RANCs could have inputs from receptor types 6–19. These networks could have some overlap in the first few layers. Overlapping RANCs with 388 inputs could produce an immense number of distinct outputs, each one correlated with a distinct odor. In addition, several RANC output cells can respond simultaneously in various combinations and at various intensities, making possible an even larger number of distinguishable odors in mixtures. Clearly, RANCs show the number of distinguishable odors is determined by such factors as selective pressure or physical limitations of sensory cells rather than any limitation in the brain's computational ability to discriminate different patterns of sensory signals.

#### Perceptual Independence of Stimulus Intensity

A biologically adaptive property of olfaction is produced by RANCs. Within certain limits, the brain separates stimulus intensity from more useful information about the source of the stimulus. Under most ordinary circumstances, the brain can identify an odor independently of its strength. A skunk's odor smells like a skunk regardless of its concentration. This perceived constancy under changes of stimulus intensity cannot result directly from the sensory receptor responses. This is because, as was pointed out earlier, receptor responses depend on the type of stimulus as well as the intensity of the stimulus: A few odorant molecules to which an olfactory receptor cell is highly sensitive can elicit the same response as many molecules to which it is only slightly sensitive. The ordering of receptor response intensities, however, does not change with stimulus intensity. By the RANC interval measure property, exactly one set of a complete n-RANC's output cells respond for each of the possible orderings of the inputs. At different concentrations, an odor is consistently identified by the set of RANC output cells that respond to it.

#### Perceptual Dependence on Stimulus Intensity

In contrast to the perceptual constancy of the preceding paragraph, the perceptions of some odors vary with the odorant concentration [13]. This is also predicted by RANCs under certain circumstances. Consider the earlier examples of the RANC correlates of the perceptions of two odors, 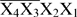 and 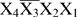, and a substance that produces the receptor response state (X_1_, X_2_, X_3_, X_4_) = (1.0, 1.0, 0.0, 0.7). As before, this state elicits responses of approximately 0.3 and 0.7 from the two RANC outputs. Since receptor type X_3_ is insensitive to this substance and X_1_ and X_2_ are saturated, X_4_ will be most affected by a change in concentration. An increased concentration that produces (X_1_, X_2_, X_3_, X_4_) = (1.0, 1.0, 0.0, 0.8) would decrease 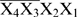 to approximately 0.2 and increase 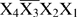 to approximately 0.8. This is the correlate of a change in the perceived strengths of the two odors.

#### Perceptual Complexity

Perceptions of mixtures provide good tests of any model because mixtures can elicit complex patterns of sensory receptor responses that result in numerous and varied perceptions that can differ markedly from the perceptions of the mixture's components. In many odorants, more than one odor can be discriminated. The perceived *complexity* of an odorant is the number of odors that can be discriminated in it. A unique odor has complexity 1.

Several studies have explored perceptions of odors in mixtures, e.g., [16]–[21]. The experiments support the conclusions that are summarized by Laing et al. [22] as four general properties, shown below. These phenomena are especially good tests of any model that attempts to explain them because they provide several detailed examples of complex perceptions. The RANC predictions of the phenomena follow from the RANC interval measure property.

1. Perceived complexity in a single odorant can be greater than in a mixture of several odorants.
  A complete n-RANC produces this phenomenon when a mixture produces fewer different receptor response intensities than the components. In the following example, substances A and B both have complexity 2, and the mixture of A and B has complexity 1. Suppose substance A elicits the olfactory receptor responses of the earlier example illustrated in Fig. 3: (X_1_, X_2_, X_3_, X_4_) = (1.0, 1.0, 0.0, 0.7). As before, the complete 4-RANC with these inputs produces correlates of two odors: 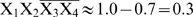 and 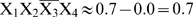. Now suppose substance B produces receptor responses (X_1_, X_2_, X_3_, X_4_) = (1.0, 0.4, 0.0, 1.0). This also produces correlates of 2 odors: 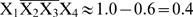 and 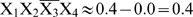. By property 5 of Table 1, a receptor's response to a mixture is at least as great as the response to any of the mixture's components. The mixture of A and B therefore produces receptor responses (X_1_, X_2_, X_3_, X_4_) = (1.0, 1.0, 0.0, 1.0). This results in only one 4-RANC response, 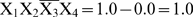, so the mixture's complexity is 1.
2. No more than three or four odors can be discriminated in mixtures.
  By the RANC interval measure property, the sum of a complete n-RANC's outputs is approximately 1. This bound on the outputs implies that not many of the outputs can be large.
3. The chemical complexity of an odorant is not correlated with the perceived complexity.
  The RANC's explanation follows from the fact that the chemical complexity of an odorant is not correlated with the number of different intensities of receptor responses. For example, several receptor types could have different sensitivities to a chemically simple substance. By the RANC interval measure property, the number of RANC outputs depends on the number of different inputs. The several different receptor response intensities produce several RANC outputs. If each output is a correlate of a perceived odor, this chemically simple substance has a large perceived complexity. Similarly, a chemically simple substance could have a small perceived complexity if it produces a small number of different receptor responses. Alternately, a chemically complex substance could have either a small or large perceived complexity, depending on whether it produces a small or large number of different receptor responses.
4. Perceived complexity is not additive when odorants are mixed.
  It is not clear why the authors listed this as a separate property, but in fact it is implied by property 1 above. The sum of the perceived complexities of two or more odorants is greater than the perceived complexity of each odorant. That is, the sum of two or more positive numbers is greater than each of the numbers. Property 1 says the perceived complexity in one of these odorants can be greater than the perceived complexity of the mixture of all of them. In that case, the sum of the perceived complexities of the components is greater than the perceived complexity of the mixture. The example in the above explanation of property 1 shows that the RANC correlate of complexity is not additive: The sum of the component complexities is 2+2 = 4, and the complexity of the mixture is 1.

### RANC Fuzzy Logic Predictions of Color Phenomena

#### The Relative Absorption Model of Color Vision

The RANCs presented in this article are a refinement and generalization of a color vision model that was recently proposed by the author [23]. That “Relative Absorption Model” (RAM) is an explicit retinal network that receives input from three classes of retinal cones and generates neural correlates of the perceptions of red, green, blue, yellow, black, and white. The RAM was shown to account for several phenomena central to color vision, such as the continuous yet categorical nature of color, mutually exclusive colors and colors that can be perceived together, color mixing, the Bezold-Brücke hue shift, the additivity failure of brightness, opponent-color cells, geometrical color space, and hue, saturation, and brightness.

**Figure 4:**
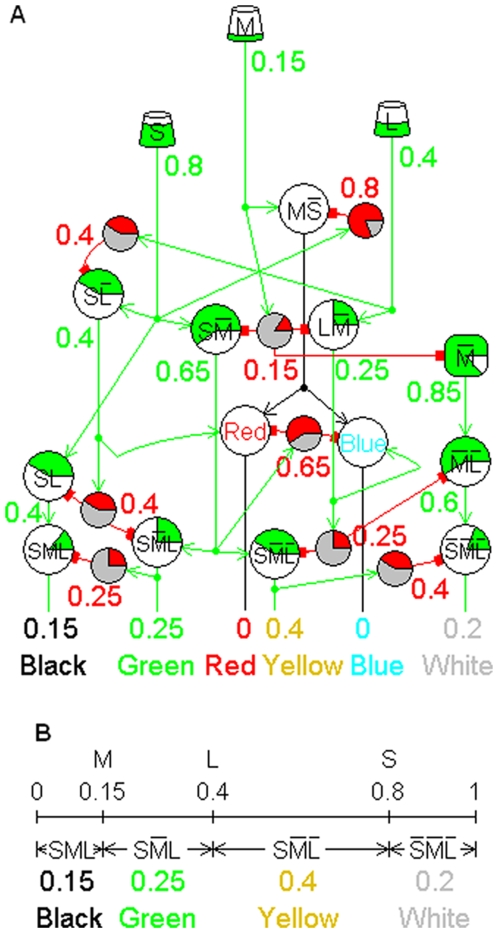
Relative Absorption Model responses to a greenish-yellow photostimulus. A greenish-yellow photostimulus moderately represses the L cone response and strongly represses the M cone so that M<L<S. The RAM's responses to a somewhat desaturated greenish-yellow photostimulus, shown in A, are correlates of the perception of the photostimulus. The graph in B shows that the approximate RAM responses illustrate the RANC interval measure property. The response 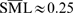 represents the fuzzy truth value of the conjunction “S and L are high and M is not high.” Since this state of cone responses is the condition for the perception of green, 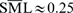 also represents the fuzzy truth value of the proposition “The photostimulus is green.” That is, 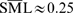 is the correlate of the perceived strength of the green component of the photostimulus.

The network in Fig. 4A is a modified 3-RANC that has inputs from the three classes of retinal photoreceptors and produces neural correlates of color vision. In the figure, S, M, and L stand for the responses of cones that are sensitive to short, medium, and long wavelengths of light. The networks for the four color cell outputs were introduced as part of the Relative Absorption Model [23]. The single 3-RANCs that produce the outputs for the black and white cells in Fig. 4A are new.

The outputs numbered 1 and 5 in Fig. 2C that are missing from Fig. 4A are 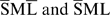, which would convey purple and violet information, respectively. A more detailed treatment of violet and purple information is given in [23]. Psychophysical evidence shows that this information is transmitted through the red and blue channels rather than through two separate channels, possibly because there was never selective pressure for the ability to obtain the complete violet and purple information. Some information is unavoidably lost in transmitting it through the red and blue channels. The combined violet and purple information is measured by 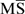, and Fig. 4A shows this response is transmitted through the red and blue channels. Except for the additional input 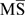 to the red and blue cells, the networks for all of the outputs in Fig. 4A are single 3-RANCs.

The RANCs were designed to form conjunctions with architectures that minimize the number of neurons required; they were not designed to fit available data about the brain. Transmitting the violet and purple information through the red and blue channels is the only exception to this design methodology, and it is only a partial exception. When it is determined that violet and purple will not have their own dedicated channels, for whatever reason, RANCs predict that the information must be transmitted through the red and blue channels. They are the only RANC channels that can convey the information without a substantial loss of information. The reasons for this are given in [23].

The new RAM in Fig. 4A is a refinement of the original in three ways. The networks for the black and white cells in Fig. 4A are RANCs. Second, these networks require fewer cells to produce the black and white outputs than the original RAM. Third, the RANC interval measure property shows that the white cell's response is a measure of how far the largest of the three cone responses is suppressed below the maximum possible response. In the original RAM, this white cell response property depended on a linear approximation of the white cell's response to its inputs.

The color names for the responses in Fig. 4A may appear to contradict the responses' logical names written on the cells. Unlike other sensory receptors, photoreceptors in vertebrates depolarize and emit transmitters spontaneously and continuously in the absence of a stimulus, and this tonic activity is suppressed by light absorption. The more light that is absorbed, the more the receptor activity is suppressed. This is the reason for property 6 of Table 1. The photoreceptors' decreasing activity function makes color perceptions somewhat counterintuitive when stated in terms of photoreceptor activity rather than photon absorption. For example, green is perceived when the M cone absorbs more photons than the S and L cones, and this high absorption suppresses the M cone activity to a lower level than the S and L responses.

The preceding section showed that RANCs can account for complex olfactory phenomena. Since RANCs also generate correlates of color vision, they predict identical color phenomena. Here it is shown that the RAM does produce neural correlates of these well-known phenomena. Because the phenomena are experienced in different sensory systems, they may not have been previously recognized as the same. How the RAM generates color phenomena may be more transparent than the RANC's explanations of olfactory phenomena because color phenomena are more familiar and because more is known about the specific photoreceptor states that produce specific phenomena. This specificity also makes more stringent demands on any model that proposes to account for the phenomena. The RAM explanations of the phenomena are the same as the RANC explanations for olfaction, except that photostimuli suppress photoreceptor activity. Except for the Bezold-Brücke hue shift, discussed immediately below, these color properties were not included in [23].

#### Effects of Stimulus Intensity on Color Perception

Color perception is largely independent of photostimulus intensity. A yellow banana appears to be yellow independently of the illuminant's intensity. The RAM's explanation of perceived color constancy is the same as the RANC's explanation for olfaction: The RAM cells that respond to a photostimulus depend on the ordering of the cones' responses, and that ordering does not change with photostimulus intensity. The cases where color perception does vary with photostimulus intensity is significant because the RAM not only predicts this can happen, but it also predicts which colors will change and in what ways. The change in color perception is known as the Bezold-Brücke hue shift [24]–[26]. Specifically, orange and greenish yellow both appear yellower at higher photostimulus intensities; violet and greenish blue appear bluer.

The RAM correlate of this effect will be explained here for greenish yellow. The RAM explanations of the other color changes are similar. A photostimulus that is perceived as greenish yellow has little effect on the S cones, suppresses the L cones somewhat, and suppresses the M cones the most of the three types. The cone response ordering is M<L<S. Unless the photostimulus is perceived to be desaturated with black or white components, there must be significant separation between the cone responses. That means the M cone is highly suppressed by the photostimulus, the S cone is only slightly suppressed or not at all, and the L cone is moderately suppressed. The L cone's mid-level response is therefore the most sensitive to changes in the photostimulus intensity. An increase in photostimulus intensity suppresses the L response closer to M and further from S. By the RANC interval measure property, this increases the RAM's yellow cell response and decrease the green cell response. That is, the RAM produces a correlate of the perception of a yellower hue at higher photostimulus intensities. Note that the photostimulus is perceived as yellower but still greenish yellow, as the RAM predicts: The RAM's green and yellow cells' response intensities change with the photostimulus intensity, but only the green and yellow color cells respond. The RAM also correctly predicts that hue shifts do not occur for unique colors. The photoreceptor response ordering for a unique yellow cell response, M = L<S, remains the same under changes in illuminant intensity. The RAM predicts a yellow banana will appear to be yellow over a wide range of illuminant intensities.

#### Perceptual Complexity

Just as more than one odor can be discriminated in an odorant, more than one hue can be perceived in a photostimulus. Red, green, blue, and yellow can each be perceived as a unique hue, meaning no other hue is perceived with it. A unique hue has complexity 1. Hues perceived as color pairs, such as greenish yellow, have complexity 2. If perceptions of black and white are counted, a photostimulus perceived as a desaturated greenish yellow could have complexity as great as 4. The RAM predicts perceptual phenomena in photostimulus mixtures that are analogous to perceptions of odors in mixtures. Perceptions of color mixtures have been explored in some detail [27], [28]. The results of these studies verify the RAM predictions.

1. Perceived complexity in a single photostimulus can be greater than in a mixture of several photostimuli.
  Color mixing experiments have shown that a greenish-yellow photostimulus superimposed on a reddish-yellow, or orange, photostimulus can be perceived as a unique yellow [27], [28]. The complexity of each of the non-unique components is greater than the unique yellow of the mixture. The RAM explanation is that a photostimulus perceived as greenish yellow elicits cone responses M<L<S. By the RANC interval measure property, the photostimulus produces a RAM correlate of greenish yellow with green and yellow cell responses. Similarly a photostimulus perceived as reddish yellow elicits cone responses L<M<S and red and yellow RAM cell responses. The mixture can produce cone responses M≈L<S and a unique yellow RAM cell response by the RANC interval measure property.
2. No more than three or four colors (including black and white) can be discriminated in mixtures.
  Color perception tests show no more than two unique colors can be perceived together – red and yellow, yellow and green, green and blue, blue and red. These color pairs can be perceived as desaturated with black and white. This bound on the number of hues that can be perceived together is predicted by the RANC interval measure property – a complete 3-RANC can have at most 4 positive outputs. Because the RANC interval measure property also implies the sum of the RAM's outputs is approximately 1, the RAM further predicts that not all four components of the color perception can be strong. When a greenish-yellow photostimulus is desaturated with both black and white components, for example, the perceived strength of at least one of these components is often so minute that identifying all four is difficult for most observers.
3. The spectral complexity of a photostimulus is not correlated with the perceived complexity.
  A monochromatic photostimulus (of a single wavelength or a narrow band of wavelengths) has the least spectral complexity. A monochromatic photostimulus with wavelength around 550 nm is perceived as greenish yellow, while a photostimulus with a complex spectral distribution can be perceived as unique yellow. This means that neither green nor red is perceived with it. The RAM accounts for this. Both the M and L cones are sensitive to wavelengths near 550 nm, but the M cone is more sensitive. The monochromatic photostimulus at 550 nm suppresses both the M and L cones, but suppresses the M cone more than the L cone, producing green and yellow RAM cell responses by the RANC interval measure property. The complex photostimulus has a balance of wavelengths that suppress the M and L cones approximately equally, producing only a yellow RAM cell response. Alternately, a monochromatic photostimulus with wavelength around 570 nm is perceived as unique yellow, while a photostimulus with a complex spectral distribution can be perceived as greenish yellow. The RAM explanation is that the M and L cones are approximately equally sensitive to the monochromatic photostimulus at 570 nm. The M and L cones are suppressed approximately equally, producing only a yellow RAM cell response. The complex photostimulus suppresses the M and L cones unequally, producing green and yellow RAM cell responses.
4. Perceived complexity is not additive when photostimuli are mixed.
  As with olfaction, property 1 implies property 4. The color example in the above explanation of property 1 shows that perceived complexity is not additive: The sum of the complexities of greenish-yellow and reddish-yellow is 2+2 = 4, and the complexity of unique yellow perceived in the mixture is 1.

### RANC Differences

The RANCs are different from most models of brain functions in several ways. They were designed to form specific logic circuits – conjunctions – with architectures that minimize a specific cost function – the number of neurons. With the partial exception for transmitting violet and purple information in the color vision model, RANCs were not designed to fit available data about the brain. In this sense, the phenomena the networks generate are genuine predictions about the brain. The RANC architectures are explicit, showing all cells and their synaptic connections; network properties do not depend on assumed “black box” capabilities of unspecified component networks or unspecified networks later in the information processing pathway. The cellular properties of excitation and inhibition are also explicitly stated; results do not depend on assumptions of sophisticated or unknown cellular capabilities.

The RANC properties given here can be proved rigorously based on the networks' architectures and the minimal cellular capabilities of excitation and inhibition; claims about network behavior do not depend on simulations that only show the demonstrated properties hold for the particular function or functions that are assumed to simulate the operation of network components. Explicit networks that generate neural correlates of known brain phenomena may explain how neurons are connected to produce those phenomena; mathematical models and networks that are not explicit cannot explain how neurons create phenomena, no matter how accurately the models might describe them. Finally, RANCs function dynamically, so their operation is consistent with the speed of most brain functions; RANC properties do not rely on any structural change, such as neurogenesis or altered synaptic connections, nor do they require any change in the way cells function, such as a change in synaptic strength or the strength of action potentials.

Most of the neurons in this article's networks show fewer synaptic connections than are typical of actual neurons. This does not necessarily mean all networks in the brain operate fundamentally differently from these networks. The networks show the simplest or nearly simplest ways neurons can be connected to perform conjunctions. For a variety of reasons, neurons in the brain may have more connections while performing the same functions in essentially the same ways. For example, the RANCs as presented here do not have redundancies or other error-correcting mechanisms. These mechanisms alone could account for much of the massive connectivity of the brain. Other reasons for multiple connections are implied by the most efficient forms of RANC architecture and will be discussed in a future paper. The purpose of this article's networks is to show that logic circuits composed of neurons can perform known brain functions. Actual networks in the brain could be organized like these minimal networks in principle while being more elaborate in the details.

### Summary and Conclusion

The Relative Absorption Model of color vision (RAM) was refined and extended here to Recursive AND NOT Conjunctions (RANCs), which are general logic circuits that perform conjunctions for the 2^n^ possible combinations of truth values of n propositions. The RANCs function dynamically, and the only neural capabilities required are excitation and inhibition. They are capable of subserving a variety of brain functions, including creative and analytical thought processes. With input from retinal cones, RANCs generate neural correlates of color vision. With olfactory receptor input, RANCs recognize patterns of signals to discriminate odors. The RANCs perform a type of fuzzy logic that has intuitive and advantageous properties, including preserving all of the information in the input signals and separating the stimulus intensity from more useful information conveyed by the stimulus, such as the identity of an odorant or spectral information about a photostimulus. The property that RANC outputs measure the intervals determined by the inputs can explain several apparently different characteristics of both color vision and olfaction.

The RANCs could have applications in other fields. Any logic circuit can be implemented with diodes and transistors, and engineers have begun to implement three-dimensional microprocessors. The architectural efficiency of three-dimensional RANCs could lead to more efficient designs of electronic processors. In a future paper, it will be shown that the most efficient forms of complete n-RANCs predict major aspects of the anatomical structure and physiological organization of the cerebral cortex.

## References

[1] Hubel DH, Wiesel TN (1959). Receptive fields of single neurons in the cat's striate cortex.. Journal of Physiology.

[2] Hubel DH, Wiesel TN (1968). Receptive fields and functional architecture of monkey striate cortex.. Journal of Physiology.

[3] Barlow H, Levick W (1965). The mechanism of directionally selective units in the rabbit's retina.. Journal of Physiology.

[4] Kupfermann I, Kandel ER (1969). Neuronal controls of a behavioral response mediated by the abdominal ganglion of Aplysia.. Science.

[5] Fried SI, Münch TA, Werblin F (2002). Mechanisms and circuitry underlying directional selectivity in the retina.. Nature.

[6] Buck L, Axel R (1991). A novel multigene family may encode odorant receptors: a molecular basis for odor recognition.. Cell.

[7] Niimura Y, Nei M (2003). Evolution of olfactory receptor genes in the human genome.. Proceedings of the National Academy of Sciences, USA.

[8] McCulloch WS, Pitts WA (1943). A logical calculus of the ideas immanent in nervous activity.. Bulletin of Mathematical Biophysics.

[9] Hopfield JJ (1984). Neurons with graded response have collective computational properties like those of two-state neurons.. Proceedings of the National Academy of Sciences.

[10] Koch C (1999). Biophysics of Computation – Information Processing in Single Neurons..

[11] Lanthorn T, Storn J, Andersen P (1984). Current-to-frequency transduction in CA1 hippocampal pyramidal cells: slow prepotentials dominate the primary range firing.. Experimental Brain Research.

[12] Rolls ET, Treves A (1998). Neural Networks and Brain Function.

[13] Kandel ER, Schwartz JH, Jessell TM (2000). Principles of Neural Science.

[14] Eggermanne BL, Serafin M, Saint-Mleux B, Bernheim L, Machard D (2003). The wake-promoting hypocretin-orexin neurons are in an intrinsic state of membrane depolarization.. Journal of Neuroscience.

[15] Zadeh LA (1962). From circuit theory to system theory.. Proceedings of the Institute of Radio Engineers.

[16] Moskowitz HR, Barbe CD (1977). Profiling of odor components and their mixtures.. Sensory Processes.

[17] Jellinek JS, Köster EP (1979). Perceived fragrance complexity and its relation to familiarity and pleasantness.. Journal of the Society of Cosmetic Chemists.

[18] Laing DG, Francis GW (1989). The capacity of humans to identify odors in mixtures.. Physiology & Behavior.

[19] Laing DG, Doty RL (1994). Perception of odor mixtures.. Handbook of olfaction and gustation.

[20] Grosch W (2001). Review. Evaluation of the key odorants of foods by dilution experiments, aroma models and omission.. Chem Senses.

[21] Wiltrout C, Dogra S, Linster C (2003). Configurational and nonconfigurational interactions between odorants in binary mixtures.. Behavioral Neuroscience.

[22] Laing DG, Doty RL, Breipohl W (1991). The Human Sense of Smell.

[23] Yoder L (2005). Relative absorption model of color vision.. Color Research and Application.

[24] Bezold W. von (1873). Ueber das Gesetz der Farbenmischung und die physiologischen Grundfarben.. Annalen der Physik und Chemie.

[25] Purdy D (1931). Spectral hue as a function of intensity.. American Journal of Psychology.

[26] Boynton RM, Gordon J (1965). Bezold-Brücke hue shift measured by color-naming technique.. Journal of the Optical Society of America.

[27] Hurvich LM, Jameson D (1955). Some quantitative aspects of an opponent-colors theory: II. Brightness, saturation, and hue in normal and dichromatic vision.. Journal of the Optical Society of America.

[28] Jameson D, Hurvich LM (1955). Some quantitative aspects of an opponent-colors theory: I. Chromatic responses and spectral saturation.. Journal of the Optical Society of America.

